# Diagnostic accuracy of point-of-care testing for acute coronary syndromes, heart failure and thromboembolic events in primary care: a cluster-randomised controlled trial

**DOI:** 10.1186/1471-2296-12-12

**Published:** 2011-03-24

**Authors:** Yuki Tomonaga, Felix Gutzwiller, Thomas F Lüscher, Walter F Riesen, Markus Hug, Albert Diemand, Matthias Schwenkglenks, Thomas D Szucs

**Affiliations:** 1 Institute of Social and Preventive Medicine, University of Zurich, 8001 Zurich, Switzerland; 2 Department of Cardiology, Cardiovascular Center, University Hospital Zurich, 8091 Zurich, Switzerland; 3 Institute for Clinical Chemistry and Haematology, Kantonsspital, 9007 St. Gallen, Switzerland; 4 FMH Foederatio Medicorum Helveticorum (Swiss Medical Association), 2800 Delémont, Switzerland; 5 Roche Diagnostics (Schweiz) AG, 6343 Rotkreuz, Switzerland; 6 European Center of Pharmaceutical Medicine, University of Basel, 4051 Basel, Switzerland

## Abstract

**Background:**

Evidence of the clinical benefit of 3-in-1 point-of-care testing (POCT) for cardiac troponin T (cTnT), N-terminal pro-brain natriuretic peptide (NT-proBNP) and D-dimer in cardiovascular risk stratification at primary care level for diagnosing acute coronary syndromes (ACS), heart failure (HF) and thromboembolic events (TE) is very limited. The aim of this study is to analyse the diagnostic accuracy of POCT in primary care.

**Methods:**

Prospective multicentre controlled trial cluster-randomised to POCT-assisted diagnosis and conventional diagnosis (controls). Men and women presenting in 68 primary care practices in Zurich County (Switzerland) with chest pain or symptoms of dyspnoea or TE were consecutively included after baseline consultation and working diagnosis. A follow-up visit including confirmed diagnosis was performed to determine the accuracy of the working diagnosis, and comparison of working diagnosis accuracy between the two groups.

**Results:**

The 218 POCT patients and 151 conventional diagnosis controls were mostly similar in characteristics, symptoms and pre-existing diagnoses, but differed in working diagnosis frequencies. However, the follow-up visit showed no statistical intergroup difference in confirmed diagnosis frequencies. Working diagnoses overall were significantly more correct in the POCT group (75.7% vs 59.6%, p = 0.002), as were the working diagnoses of ACS/HF/TE (69.8% vs 45.2%, p = 0.002). All three biomarker tests showed good sensitivity and specificity.

**Conclusion:**

POCT confers substantial benefit in primary care by correctly diagnosing significantly more patients.

**Trial registration:**

DRKS: DRKS00000709

## Background

Chest pain, tightness, pressure or squeezing, along with dyspnoea and heartburn-like sensations, are a diagnostic challenge in primary care medicine. Symptoms are often an inadequate guide to a working diagnosis. Common causes of chest pain and dyspnoea include stable angina, gastrointestinal disease, panic disorder, viral infection and musculoskeletal pain, but the most serious suspects include acute coronary syndromes (ACS), heart failure (HF) and thromboembolic events (TE) [1-3].

Numerous cardiovascular biomarkers are now available [4]. Cardiac troponin T (cTnT), N-terminal pro-brain natriuretic peptide (NT-proBNP) and D-dimer are the most used, in particular for cardiovascular risk stratification [5]. New multifunctional devices measure all three in minutes.

cTnT is a highly specific and sensitive protein for diagnosing myocardial necrosis: elevation diagnoses ACS and identifies patients at high risk of cardiac events [6]. NT-proBNP differentiates cardiac from non-cardiac causes of dyspnoea and excludes HF in symptomatic patients [7]. As reported by Jernberg et al. [8], NT-proBNP analysis improves the early detection of patients with potential ACS and non ST-segment elevation myocardial infarction (NSTEMI). NT-proBNP elevation also has high prognostic value, being associated with increased mortality in cardiovascular patients [9]. The strength of D-dimer, an indicator of fibrin degradation and coagulation activation, lies in its high negative predictive value (NPV) for excluding TE (deep vein thrombosis [DVT] and pulmonary embolism [PE]) [10]. Its specificity is usually low since increased levels are encountered in many non-thrombotic situations [11].

Our aim was to analyse the benefit of POCT for cardiovascular risk stratification in primary care. We hypothesised that POCT testing for cTnT, NT-proBNP, and/or D-dimer in venous whole blood would allow for a more accurate diagnosis of ACS, HF and TE by office-based, Swiss general practitioners.

## Methods

### Patients and study design

We randomised 68 primary care practices in Zurich County (Switzerland) to diagnostic aid from a POCT analyser (n = 33 [39 physicians]; POCT group) or to conventional diagnosis employing best clinical practice (n = 35 [40 physicians]; controls). We randomised only practices at least 8-10 km from Zurich and Winterthur where laboratories with specialised diagnostic systems are less available.

All patients presenting with potentially cardiovascular chest pain or symptoms between May 2006 and August 2007 were invited to participate. Patients gave written informed consent and the study received approval from the cantonal ethics committee (Kantonale Ethikkommission Zürich), in line with the Declaration of Helsinki (1996) and Good Clinical Practice guidelines. Non-inclusion criteria were refusal of consent, presentation >5 days after symptom onset, recent anticoagulant treatment, severe renal dysfunction and cancer therapy. Rationales for the exclusion criteria were the normalisation of the cTnT level five days after ACS, the fact that cTnT may be increased even in the absence of clinically suspected acute myocardial ischemia in patients with renal insufficiency, and the unpredictable effect of anticoagulant treatment and cancer therapy on the biomarkers' concentration [12,13].

Physicians examined all patients before making a working diagnosis of ACS, HF, TE, musculoskeletal or "other" (specified) problems based on the patients characteristics, medical history, symptoms, physical findings and, in the POCT group, the biomarkers (it was not mandatory to analyse all biomarkers: the physicians had the possibility to choose if and which biomarker test was necessary). At follow-up 3 weeks later the same physician reviewed the working diagnosis. The follow-up diagnosis was defined as the confirmed diagnosis. Follow-up data of patients requiring additional specialist visits or hospitalisation were provided by specialist or hospital reports.

### Technical information

POCT practices received a bedside Cardiac Reader^® ^(Roche Diagnostics, Switzerland), a 3-in-1 device that determines cTnT, NT-proBNP or D-dimer in heparinised venous whole blood within 8-12 min. Measurement was quantitative for each parameter over the ranges 0.05-2.00 ng/ml, 60-3000 pg/ml and 0.1-4.0 μg/ml, with validated positive/negative cut-offs of 0.1 ng/ml, 125 pg/ml and 0.5 μg/ml. Instruction to the POC-instrument always was performed by the same specialist from Roche Diagnostic. All GPs who received a POCT device received advice on the interpretation of test results. Test quality was monitored using the internal and external quality controls required by Swiss federal law and the Swiss Commission for Quality Assurance in the Medical Laboratory (QUALAB) [14].

### Statistical analysis

Data were analysed using SPSS 14.0 for Windows. Intergroup comparisons of categorical data were performed using univariate logistic regression; standard errors and p values were adjusted for the effect of clustering utilising a generalised estimating equations approach. We detected an interclass correlation coefficient of 0.073 for the main binary endpoint of a correct versus incorrect working diagnosis.

To evaluate diagnostic test quality and performance we plotted receiver operating characteristic (ROC) curves, defined as plots of test sensitivity on the y axis vs. 1-specificity on the × axis. The area under the ROC curve (AUC) combining sensitivity and specificity was used to measure overall diagnostic test performance and was interpreted as the average sensitivity value for all possible specificity values.

## Results

### Study population

Of the 369 patients recruited, 218 (59%) were enrolled in the POCT group (7 ± 10/practice) and 151 (41%) in the control group (4 ± 4/practice). Characteristics and blood parameters were similar in both groups (Table 1).

**Table 1:** Baseline demographics, clinical chemistry and interval between symptom onset and baseline presentation.

Variable	POCT (n = 218)	Controls (n = 151)
	n (%) or mean ± SD	n (%) or mean ± SD
Men	121 (57.9)	83 (58.0)
Age [years]	65 ± 16	64 ± 17
Weight [kg]	80 ± 17	78 ± 17
Height	169 ± 10	169 ± 9
Body mass index [kg/m^2^]	28 ± 5	27 ± 6
Glucose [mmol/l]	5.9 ± 1.5	6.3 ± 2.1
Creatinine [mmol/l]	84.4 ± 29.1	84.4 ± 22.4
High-density lipoprotein [mmol/l]	1.4 ± 0.7	1.4 ± 0.5
Low-density lipoprotein [mmol/l]	2.9 ± 1.0	3.3 ± 1.0
Triglycerides [mmol/l]	1.7 ± 1.1	1.7 ± 1.0

Interval between symptom onset and baseline visit [all patients, days]	3.3 ± 9.8	1.7 ± 1.7
Interval between symptom onset and baseline visit [patients presenting <5 days after symptom onset, days]	1.2 ± 1.4 (n = 185)	1.5 ± 1.4 (n = 141)

POCT, point-of-care testing; SD, standard deviation.

The interval between symptom onset and baseline visit was much longer in the POCT group because some (mainly POCT) patients presented over 5 days after symptom onset (non-inclusion criterion). We contacted the practices for specific explanations. In almost all cases the patients had visited the physician in the previous weeks/months (> > 5 days) with similar problems. Due to new or exacerbated symptoms (onsetting in the previous 5 days) they revisited their physician who reported the date of their first or previous visit incorrectly. After patients with incorrect symptom onset were excluded, the average interval between symptom onset and baseline visit became similar in the two groups.

### Presenting symptoms

Many patients reported mixed sensations of pain or discomfort. Both groups had high prevalence of acute chest pain, tightness, pressure or squeezing, dyspnoea and heartburn-like sensations. Other symptoms were relatively rare (Table 2). The groups statistically differed in rates of acute chest and calf pain.

**Table 2:** Presenting symptoms.

Symptoms	POCT (n = 218) n (%)	Controls (n = 151) n (%)
Acute chest pain	99 (45)	96 (64)
Tightness, pressure, or squeezing in the chest	138 (63)	92 (61)
Dyspnoea	99 (45)	69 (46)
Heartburn-like sensation	32 (15)	19 (13)
Heaviness and tension sensations in the leg	16 (7)	5 (3)
Calf pain	16 (7)	4 (3)
Neck vein congestion	4 (2)	4 (3)
Oedema	22 (10)	9 (6)
Nocturia	7 (3)	4 (3)
Cyanosis	2 (1)	3 (2)

### Medical history

The most frequent pre-existing diagnoses were hypertension, diabetes and angina. Only the proportion of patients with previously diagnosed HF differed substantially between the groups (Table 3). Pathological electrocardiograms (ECGs) were more prevalent in controls. Reasons for hospitalisation in the previous 12 months were diverse and only partially cardiovascular.

**Table 3:** Medical history.

	POCT (n = 218) n (%)	Controls (n = 151) n (%)
Malignant tumour	15 (7)	7 (5)
Thrombosis/embolism	12 (6)	13 (9)
Myocardial infarction	15 (7)	11 (7)
Heart failure	19 (9)	5 (3)
Angina	20 (9)	15 (10)
Renal failure	13 (6)	8 (5)
Diabetes	34 (16)	15 (10)
Aneurysm/dissection	1 (1)	2 (1)
Hypertension	48 (22)	23 (15)
Chronic obstructive pulmonary disease	9 (4)	5 (3)
Peripheral arterial disease	10 (5)	5 (3)
Smokers	35 (16)	24 (16)
Pathological electrocardiogram	71 (33)	59 (39)
Hospitalisation in previous 12 months	43 (20)	25 (17)

### Working and confirmed diagnoses

**Figure 1:**
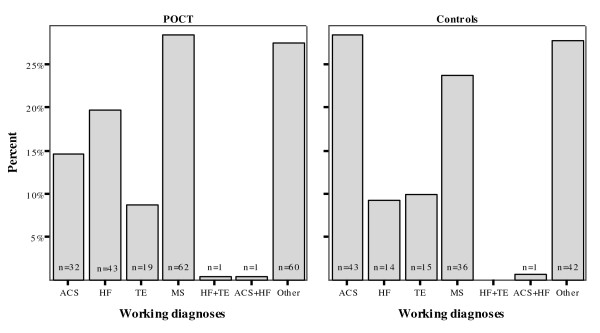
**Working diagnoses at baseline**. ACS diagnoses were more frequent, and HF diagnoses less frequent, in controls (p = 0.03 and p = 0.04). TE diagnoses were similar in both groups (p = 0.81). One patient per group was diagnosed with both ACS and HF, and one POCT patient with both HF and TE. Most diagnoses in both groups were musculoskeletal or "other" problems (p = 0.35 and p = 0.97). (ACS, acute coronary syndromes; HF, heart failure; MS, musculoskeletal problems; TE, thromboembolic events).

Some working diagnoses differed in frequency between the groups (Figure 1). In the POCT group ACS was less frequent, and HF more frequent, than in controls (p = 0.03 and p = 0.04). TE was similar in frequency (p = 0.81), as were musculoskeletal problems (p = 0.34) and "other" diagnoses (e.g. stable angina, pleuritis, psychiatric problems, viral infection; p = 0.97).

**Figure 2:**
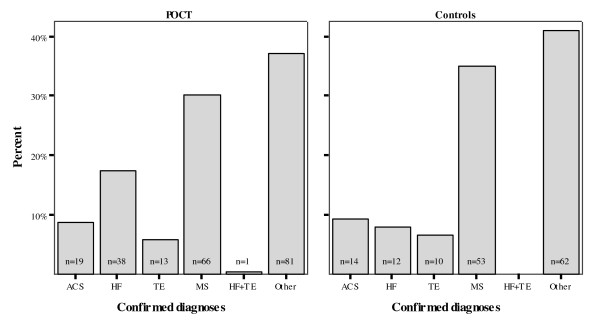
**Confirmed diagnoses**. The incidences of ACS and TE were similar in both groups (p = 0.87 and p = 0.93). HF appeared more frequent in the POCT group but the difference was nonsignificant (p = 0.08). Musculoskeletal and "other" problems were the predominant diagnoses (p = 0.49 and p = 0.57). (ACS, acute coronary syndromes; HF, heart failure; MS, musculoskeletal problems; TE, thromboembolic events).

However, confirmed diagnoses did not differ in frequency (Figure 2). Intergroup ACS and TE were similar (p = 0.87 and p = 0.93), while HF remained substantially, but non-significantly, more frequent in the POCT group (p = 0.08). In both groups, two-thirds of patients had musculoskeletal problems (p = 0.49) or "other" diagnoses (p = 0.57).

### Diagnostic accuracy of the working diagnosis

In the POCT group 165/218 working diagnoses (76%) proved correct vs 90/151 (60%) in controls (p = 0.002). Seventy percent of the working diagnoses of ACS, HF and TE proved correct in the POCT group vs 45% in the controls (p = 0.002), with false-positives in 29 (30%) patients vs 40 (55%) patients, respectively. In contrast, correctness of the remaining working diagnoses (musculoskeletal or "other" problems) did not differ: 80% in the POCT group vs 73% in the controls (p = 0.31).

Except for ACS, where sensitivity was higher in controls, working diagnoses were more sensitive in the POCT group (Table 4). However, specificity was similar in both groups, except for ACS, where it was higher in the POCT group. NPVs of the ACS, HF, and TE diagnoses were near-identical. Overall these results confirmed greater diagnostic accuracy in the POCT group.

**Table 4:** Diagnostic accuracy of the working diagnoses - sensitivity, specificity and NPV.

		Sensitivity		Specificity		NPV	
		**n**	**%**	**n**	**%**	**n**	**%**
ACS	POCT	17/19	90	183/199	92	183/185	99
	Controls	14/14	100	107/137	78	107/107	100

HF	POCT	39/39	100	173/179	97	173/173	100
	Controls	10/12	83	134/139	96	134/136	99

TE	POCT	14/14	100	198/204	97	198/198	100
	Controls	9/10	90	135/141	96	135/136	99

MS	POCT	54/66	82	144/152	95	144/156	92
	Controls	34/53	64	96/98	98	96/115	84

Other	POCT	55/81	68	132/137	96	132/158	84
	Controls	37/62	60	84/89	94	84/109	77

(i) ACS, acute coronary syndromes; HF, heart failure; MS, musculoskeletal problems; NPV, negative predictive value; TE, thromboembolic events.

### Biomarker performance in the POCT group

Individual test sensitivities and NPVs were generally higher when assessed using the confirmed diagnoses. Specificities, in contrast, differed only marginally.

The diagnostic power of the cTnT test was higher when assessed using the confirmed diagnoses: sensitivity and NPV were 17% and 6% higher (Table 5). Seven patients had a false-negative result and risked a wrong diagnosis. Two had a history of myocardial infarction and were polymedicated (statins, aspirin, diuretics, β-blockers); based on their history and medication they were correctly diagnosed with ACS. Another had a history of angina and was polymedicated: recurrent angina was suspected. A patient presenting 5 hours after symptom onset was diagnosed with stable angina. The final three patients were diagnosed correctly from their symptoms.

**Table 5:** Sensitivity, specificity and NPV of cardiovascular biomarkers in relation to the working and confirmed diagnoses.

Biomarker	Working/Confirmed diagnosis	Sensitivity		Specificity		NPV	
		
		n	%	n	%	n	%
cTnT	Working ACS	11/26	42	113/121	93	113/128	88
	Confirmed ACS	10/17	59	121/130	93	121/128	95

NT-proBNP	Working HF	33/35	94	26/35	74	26/28	93
	Confirmed HF	31/31	100	28/39	72	28/28	100

D-dimer	Working TE	16/20	80	78/98	80	78/82	95
	Confirmed TE	13/14	93	81/104	78	81/82	99

(i) ACS, acute coronary syndromes; cTnT, cardiac troponin T; HF, heart failure; NPV, negative predictive value; NT-proBNP, N-terminal pro-brain natriuretic peptide; TE, thromboembolic events.

The diagnostic power of the NT-proBNP tests was high: all patients with HF also had a positive NT-proBNP, and while none of those with a negative NT-proBNP had HF. Specificity was 72%.

The D-dimer test had a sensitivity of 93%, specificity of 78% and NPV of 99%. The single false-negative was in a 65-year-old man with a history of cancer who was correctly diagnosed with deep vein thrombosis from his symptoms.

**Figure 3:**
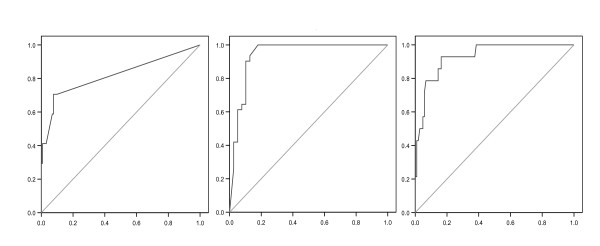
**cTnT (left), NT-proBNP (centre) and D-dimer (right) ROC curves**. X-axis: 1-Specificity; Y-axis: Sensitivity. Areas under the curve (AUC): 82% (95% confidence interval [CI]: 0.69-0.95), 94% (95% CI: 0.88-1.0) and 93% (95% CI: 0.87-0.99).

The cTnT ROC curve based on the confirmed diagnoses was somewhat oddly shaped because physicians often recorded the result simply as positive or negative (< 0.1 ng/ml). In such cases positive and negative cTnT values were arbitrarily entered as 0.1 ng/ml and 0.0 ng/ml. Very high test specificity accounted for the measured AUC, 82% (Figure 3). The NT-proBNP and D-dimer ROC curves were more regular, achieving high sensitivity and specificity, with AUCs of 94% and 93%.

## Discussion

Our results emphasise the clinical value of POCT using a novel cardiovascular marker device measuring cTnT, NT-proBNP and D-dimer for the risk stratification of ACS, HF and TE in primary care. Diagnoses were more accurate in the POCT group. In particular, POCT-assisted physicians avoided numerous false-positive ACS, HF, and TE diagnoses. This evidence supports the use of cTnT, NT-proBNP and D-dimer in diagnosing patients with chest pain or other potentially cardiovascular symptoms. Analysis of these biomarkers provides clinical benefit by more accurately ruling in/out ACS, HF and TE, each with significant management implications.

### ACS diagnosis

In previous studies, atypical or absent chest pain has been associated with failure to recognise ACS by either physician or patient in up to one third of cases [15]. Our study shows a reverse tendency: the number of working diagnoses of ACS was unexpectedly high, in particular in controls. Perhaps the study topic alerted physicians to ACS as a possible cause of chest pain, causing them to overestimate its incidence, in particular in the unassisted control group. Many working diagnoses were overturned on follow-up, resulting in very high sensitivity and low specificity. In contrast, the POCT group suspected fewer ACS, resulting in high sensitivity and specificity. Despite two false-negatives in patients wrongly suspected of having stable angina, POCT was more accurate in stratification: ACS was correctly ruled in/out in 92% of POCT patients vs 78% of controls.

Overall, these findings support the notion that when combined with clinical symptoms and ECG screening, troponin assay improves diagnostic accuracy, in line with American College of Cardiology/American Heart Association guidelines on managing patients with unstable angina or NSTEMI [16]. Except for the sensitivity results discussed above, our data reflect the 94% sensitivity and 97% NPV of the cTnT test for ACS reported by Lüscher et al. [17] in 92 patients with chest pain, and the 100% sensitivity, 42% specificity and 100% NPV reported by Fehr et al. [18] in a cross-sectional study of asymptomatic haemodialysis patients. In our study, the high AUC value of the cTnT ROC curve, a measure of overall quality, was largely due to the very high test specificity. To rule out ACS on the basis of cTnT, a minimum of 5 hours (time to increase above the reference range) and a maximum of 5-7 days (time for biomarker level normalisation) must normally elapse after symptom onset. Table 1 confirms that most patients complied with these time limits.

The cTnT assay itself yielded seven false-negatives. Most patients concerned (5/7) were correctly diagnosed from their history and symptoms. Recurrent angina was suspected in the other two patients. High drug consumption in three patients, and the short interval (5 h) between symptom onset and presentation in one patient, may partially explain the false-negatives. Misuse of the POCT device may account for the other cases. That the same practice made 4/7 false-negative diagnoses supports this explanation, highlighting the importance of adequate training on POCT devices. In our study, training on the POCT instrument always was performed by the same Roche Diagnostics specialist, but some staff members were instructed by their colleagues only.

### HF diagnosis

HF is a common and increasing public health problem [19]. Diagnosis in the primary care setting can be difficult and incorrect in up to 70% of cases [20]. Echocardiography is the gold standard for identifying and confirming left ventricular systolic dysfunction [21] but is relatively expensive and generally beyond the resources of general practitioners [22,23]. Even where available, its appropriate use requires extensive training and experience.

In our study, diagnosis of HF in the POCT group showed high sensitivity, specificity and NPV. Sensitivity in the controls was relatively low, but both specificity and NPV were high. These results indicate that HF was better identified in the POCT group, presumably because of the NT-proBNP data. Assay sensitivity and NPV were perfect: all patients with HF had elevated NT-proBNP values, whereas HF was correctly excluded in all patients with normal values. The very high AUC of the NT-proBNP ROC curve (Figure 3) reflects the test's high diagnostic accuracy. These results confirm earlier studies: the NZ prospective *Natriuretic Peptides in the Community Study*, a randomised controlled trial of the effect of NT-proBNP on HF diagnostic accuracy in primary care, showing significant improvement in the BNP group over controls [24]; a UK study in 306 primary care referrals for suspected HF emphasising the high NPV of the NT-proBNP assay (ECG had no additional predictive value) [25]; and the prospective 600-patient *N-terminal PRo-BNP Investigation of Dyspnea in the Emergency department *(PRIDE) study which found the test with its 99% NPV to be a valuable addition to standard clinical assessment for identifying and excluding acute HF: a positive NT-proBNP was the strongest independent predictor of a final positive diagnosis of HF.

### TE diagnosis

TE includes PE and DVT, which are closely interrelated since 90% of symptomatic PE arise from leg vein thrombi [26]. It's well recognised that TE cannot unequivocally be diagnosed from the history and physical examination alone, even in high-risk patients [27]. The recent development of non-invasive D-dimer blood tests with a very high NPV has markedly enhanced the accuracy of diagnosis [28].

In our study, the sensitivity, specificity and NPV of the TE diagnoses were good in both groups, making it difficult to determine whether diagnoses in the POCT group were more accurate. The D-dimer assay showed moderate specificity but very high sensitivity and NPV. The only patient with a false-negative D-dimer was a 65-year-old man with a history of cancer who was correctly diagnosed with a DVT based on his symptoms. The NPV confirmed the assay's power in excluding TE. While a negative D-dimer safely excludes TE, patients with a positive result should still be screened for PE and DVT, in particular in the presence of typical symptoms. Our results are consistent with those of other studies, e.g. Leclercq et al. or Schutgens et al. [29,30].

### Study limitations and future research

Some limitations of the study require discussion. For example, we confined ourselves to Zurich County for practical reasons; whether the results can or cannot be extrapolated to less urbanised areas remains to be determined.

A second limitation was patient recruitment. Initially, recruitment was clearly higher in the POC group. We put this down to a difference in study awareness: the device on their premises made POCT physicians more study-aware. Control physicians, on the other hand, had only the study protocol and the questionnaires to remind them, and perhaps forgot to recruit; after we initiated regular telephone reminders, recruitment rates became almost equal in both groups.

The follow-up diagnoses were another limitation. In principle, they should have been performed by independent blinded assessors but this was not feasible. For practical and data protection reasons, they were made by the same physician who was primarily consulted by the patient. We are aware of the potential for bias, e.g. due to possible underreporting of incorrect baseline diagnoses leading to a false high rate of correct baseline diagnoses or a false low difference between study arms. On the other hand, for patients referred for further diagnostic work-up (including all patients at potentially high cardiovascular risk), GPs received a written report on the second-stage assessment as well as information on further clinical management, which substantially reduced the risk of bias.

A fourth limitation concerns possible underestimation of the number of HF diagnoses in the controls. Not only were there significantly more pre-existing HF diagnoses in the POCT group, but the total number of newly identified HF patients was also greater. We assume that the POCT physicians used the NT-proBNP value as diagnostic criterion to identify more patients with NYHA class I-II HF. Indeed, early stages of HF are often asymptomatic and difficult to identify without accurate screening. Some patients with mild HF may not have been diagnosed as a result, and this tendency would have been stronger in the controls.

As shown in Table 5 which compares biomarker performance in the POCT group to the working and confirmed diagnoses, it seems that in some cases the GP decided to ignore the results of the biomarker tests. It is unknown if there was a lack of confidence in the biomarkers, if there was a problem with the interpretation of the results, or if GPs gave more importance to physical findings, symptoms, and patient history. In any case, this finding emphasises the importance of training and continuing medical education.

Health-economic and cost-effectiveness studies of cardiovascular risk stratification using POCT are rare. Some studies have analysed the costs and benefits of a single biomarker for a defined disease. Thus Nielsen et al. [31] concluded that NT-proBNP testing in primary care patients with dyspnoea halved the need for echocardiographic screening. Gustafsson et al. [32] reported that a normal NT-proBNP effectively ruled out left ventricular systolic dysfunction in primary care patients referred for echocardiography, thereby avoiding unnecessary further investigation. Siebert et al. [33] found that NT-proBNP measurement improved patient outcome, reduced echocardiography use by 58%, prevented 13% of hospitalisations and reduced hospital stay by 12%. Similar studies have been performed for other biomarkers, but a more general evaluation is needed of the possible benefits of POCT for ACS, HF and TE in primary care.

## Conclusions

This study found substantial benefit for POCT diagnosis in cardiovascular risk stratification at the primary care level. Non-invasive analysis of cTnT, NT-proBNP and D-dimer produced more accurate diagnoses of ACS, HF and TE in the POCT group. Given the potential for substantial health-economic savings, we plan a fuller investigation based on the clinical outcomes of this study.

## Competing interests

Prof. Felix Gutzwiller and Albert Diemand own some Hoffmann-La Roche stock. Albert Diemand declares a financial conflict, since Roche Diagnostics (Switzerland) provided all POCT instruments, reagents, and an unrestricted educational grant. However, he had no access to the dataset and no influence on the data analysis performed at the Institute of Social and Preventive Medicine, Zurich University. All other authors have no conflicts of interest.

## Authors' contributions

All authors contributed to the conception and design; YT undertook the statistical analysis and MS advised on the analysis of clustered-randomised data; all authors contributed to the interpretation of data and have been involved in the drafting of the manuscript or revising it critically for important intellectual content; and have given final approval of the version to be published.

## Pre-publication history

The pre-publication history for this paper can be accessed here:

http://www.biomedcentral.com/1471-2296/12/12/prepub
